# Assessing satisfaction in simulation among nursing students: psychometric properties of the Satisfaction with Simulation Experience - Italian Version scale

**DOI:** 10.1186/s12912-024-01974-1

**Published:** 2024-04-30

**Authors:** Sara Alberti, Massimo Guasconi, Marina Bolzoni, Giulia Donnini, Paola Volpi, Sergio Rovesti, Federico Monaco, Antonio Bonacaro, Paola Ferri

**Affiliations:** 1 Clinical and Experimental Medicine PhD Program, Department of Biomedical, Metabolic and Neural Sciences, University of Modena and Reggio Emilia, str. Giuseppe Campi n° 287, Modena, 41125 Italy; 2 Department of Medicine and Surgery, University of Parma, via Gramsci n° 14, Parma, 43126 Italy; 3 Azienda USL of Piacenza, via Taverna 49, Piacenza, 29121 Italy; 4 University Hospital Polyclinic of Modena, via del Pozzo 71, Modena, 41124 Italy; 5 Department of Biomedical, Metabolic and Neural Sciences, University of Modena and Reggio Emilia, str. Giuseppe Campi n° 287, Modena, 41125 Italy

**Keywords:** Simulation, Student satisfaction, Instrument Validation, Nursing students, Teaching and learning, Psychometrics

## Abstract

**Background:**

The Satisfaction with Simulation Experience scale is a 5-point Likert scale that measures students’ satisfaction in medium and high-fidelity simulation scenarios. This study aims at investigating the psychometric properties of the Satisfaction with Simulation Experience - Italian Version scale.

**Methods:**

A multi-centre cross-sectional study was conducted. The scale was administered to a sample of 266 undergraduate nursing students from two Italian universities after attending a medium- and high-fidelity simulation session in November 2022 and March 2023. Cronbach’s alpha coefficient and item-total correlation were sorted out to assess internal consistency and reliability. The test-retest method was used as a measure of scale stability over time as well as the confirmatory factor analysis to verify construct validity.

**Results:**

The Cronbach’s alpha value was 0.94 for the overall scale, indicating excellent reliability, and it was 0.84 or higher for each subscales, indicating good reliability. A large correlation coefficient of 0.60 or higher was found between each item and its subscale and between each item and the overall scale score. A medium test-retest correlation coefficient was found for most items (*r* > 0.30). The confirmatory factor analysis confirmed the factorial structure found in the original study.

**Conclusions:**

Satisfaction is an important teaching and learning quality indicator along with the achievement of learning outcomes in simulation. The Satisfaction with Simulation Experience - Italian Version scale showed good reliability and validity; therefore, it could be a useful tool to assess simulation impact in Italian nursing students. The extensive utilization of the Satisfaction with Simulation Experience scale, along with its various validated versions, could facilitate assessing satisfaction in simulation across diverse contexts and enable comparisons of findings across studies in different countries.

## Background

Current evidence highlights the potential power of simulation as a technology-based educational strategy in promoting better learning outcomes in students and professionals in the healthcare field [1]. Simulation is defined as “the process by which we are trying to achieve results approximating clinical practice as closely as possible” [2]; it is an educational strategy rather than a technology [2, 3], through which students may experience real-world elements that are observable and therefore assessable by teachers [4, 5].

Following the covid-19 pandemic [6, 7], and the integration of state-of-the-art technologies [1] the use of simulation in healthcare professionals’ education has been increasing significantly. Furthermore, simulation has been recognised as a key strategy for acquiring essential skills to work in unpredictable and complex environments where mutual dependence and cooperation with other professions are vital in delivering high-quality care [8, 9].

Moreover, literature reviews show that simulation improves knowledge and skills among undergraduate health students [1, 7, 10]. Additionally, simulation contributes in reducing anxiety and stress and in fostering reflective learning, self-confidence and satisfaction [11–15].

Despite being recognised as an important variable, satisfaction alone does not provide a full picture of the effectiveness of simulation [16]. In the field of social science, students’ learning satisfaction is defined as the impact of the process which have taken place during a teaching and learning experience. Thus, it may play a crucial role in fostering students’ willingness to continue studying in a life-long perspective and promote learning outcomes achievement [17]. Several literature reviews reported how students’ satisfaction is related to simulation [7, 11, 18–21]. The majority of nursing students showed a high level of satisfaction with simulation [22, 23] and qualitative evaluation also revealed that students generally have positive perceptions of their simulation experiences [23, 24]; however, evidence is inconsistent when compared with traditional methods [25]. Student satisfaction is greater in high-fidelity simulation than in virtual learning [26] and it increases after repeated exposure to it [27]. High-fidelity simulation achieves higher levels of satisfaction in comparison to low-fidelity simulation or paper-based case study activities [28]; in contrast, this is not the case when compared with medium-fidelity simulation [29]. Furthermore, in their meta-analysis Yi Li et al. (2022) reported that high-fidelity simulation is not likely to increase learning satisfaction in nursing students instead it could prove to be more beneficial when compared with other teaching methods. This finding may be due to simulation-related factors. Consequently, authors concluded that nursing educators are required to implement evidence-based strategies aimed at improving students’ learning satisfaction [21]. Learners’ satisfaction in simulation can be assessed in a variety of ways, both qualitative and quantitative [16]. Typically, students are asked to respond to a survey based on Likert-type questions.

In 2011, Levett-Jones et al. developed and validated the Satisfaction with Simulation Experience (SSE) scale, a tool designed to compare differences in satisfaction levels in undergraduate nursing students exposed to medium and high-fidelity simulation sessions in Australia [3]. The SSE scale is based on a reflective model and it consists of three sub-scales [30]. The scale has recently been validated in other countries, including Italy [31], Croatia [32] and Turkey [33] and it was evaluated among healthcare professionals from various disciplines, as well as post-graduate healthcare course students [6, 30, 32, 34–36].

The validation process is paramount to ensure that the tool is accurate and reliable [37]. Accurate translation is crucial for the validation process, ensuring the tool aligns with the cultural and linguistic nuances of a different geographical setting.

In fact, the delivery of high-quality educational interventions depends on the accurate assessment and deeper understanding of an individual’s cultural and linguistic background [38].

In Italy, a first validation study of the Satisfaction with Simulation Experience - Italian Version (SSE-ITA) scale was carried out on a sample of 10 undergraduate nursing students which included a content validity assessment [31]. However, as authors reported, a greater sample size was needed to confirm psychometric integrity of the newly validated tool. Furthermore, the research team recommended testing the tool in different contexts and cohorts of students with the aim of producing further evidence of reliability and construct validity [31].

Therefore, this study aims at investigating the psychometric properties of the SSE-ITA scale on a larger sample.

## Methods

A multi-centre cross-sectional study was carried out in 2022–2023 to test the psychometric properties of the SSE-ITA scale among Italian undergraduate nursing students.

### Sampling and data collection

A convenience sample of nursing students from two Italian universities was recruited. Specifically, students enrolled in the third year of the Bachelor of Science in Nursing at the University of Modena and Reggio Emilia and first-year students at the University of Parma, who took part in at least one simulation session scheduled in Academic Year 2022–2023, were voluntarily recruited.

Students of the University of Modena and Reggio Emilia filled out the SSE-ITA scale following a high-fidelity simulation session delivered in October and November 2022. The test-retest reliability of the SSE-ITA scale was assessed by administering it to the sample at the end of the simulation session and additionally, on average, 8 days later than simulation session (range 4–42 days). Students of the University of Parma filled out the SSE-ITA scale after taking part in a medium-fidelity simulation session arranged in March 2023.

### Tool

The SSE-ITA scale aims at assessing nursing students’ satisfaction following a high or medium-fidelity simulation session.

As in the original version of the scale [3], the Italian one is based on a set of 3 sub-scales consisting of 18 items exploring different areas of the simulation experience [30] associated to 5-point Likert scale statements (Strongly Disagree, Disagree, Not Sure, Agree, Completely Agree).

The 3 above-mentioned sub-scales focus on the following areas:

- Sub-scale 1 titled “Debriefing and Reflections” consisting of 9 items explores participants’ opinions on opportunities for reflection and learning at debriefing stage;
- Sub-scale 2 titled “Clinical Reasoning’’ consisting of 5 items assesses the effectiveness of simulation in fostering clinical reasoning skills;
- Sub-scale 3 titled “Clinical Learning,” consisting of 4 items assesses to what extent simulation supports clinical skills development.

In the first Italian validation study, the SSE-ITA showed an Item-Content Validity Index value (I-CVI) ≥ 0.80 and a Subscale-Content Validity Index (S-CVI) equal to 0.94. The reliability coefficient (r) was 0.88 and internal consistency values (Cronbach’s alpha) for each sub-scale were: ‘’Debriefing and reflections’’ α = 0.74; ‘’Clinical reasoning’’ α = 0.69; ‘’Clinical learning’’ α = 0.63; overall scale = 0.71 [31].

### Simulation sessions

High-fidelity simulation sessions were delivered at the Centre for Advanced Training and Medical Simulation of the University of Modena and Reggio Emilia. The students were divided into ten groups. Each group participated in a high-fidelity simulation session between October and November 2022. The simulation session was conducted by an experienced simulation instructor and structured as follows: briefing (1 h), simulation session (40 min), and debriefing (1 h). The scenario was based on a deteriorating patient in the emergency department. The patient’s clinical conditions were aimed at pointing out that the patient was about to go into cardiac arrest. Once cardiac arrest was recognised and confirmed, students had to perform Basic Life Support and Defibrillation (BLSD) according to current guidelines [39]. The expected learning outcomes were: the application of the National Early Warning Score (NEWS) scale [40] and of the BLSD algorithm, the correct prioritisation of interventions in accordance with the resources available along with effective communication among team memebers.

University of Parma students were involved in medium-fidelity simulation session focusing on head-to-toe standardised clinical examination based on ABCDE algorithm, on sorting out the NEWS score and on reporting clinical conditions for further care via the SBAR tool [41]. The activity took place in the SIMLAB of the Department of Medicine and Surgery. The same structured approach used in the Modena and Reggio Emilia centre was adopted in Parma. The simulation session was delivered by an experienced simulation instructor and included: briefing (1 h), simulation session (40 min), and debriefing (1 h). Students were divided into 29 groups of 5 students each.

The expected learning outcomes were: appropriate patient assessment through head-to-toe clinical examination, correct application of the NEWS 2 scale, and the effective use of the SBAR tool.

### Data analysis

The item/participant ratio equal to or greater than 10:1 was considered to define the sample size according to the indications of Costello & Osborne, 2005 [42]. The characteristics of the sample (gender and age) were analysed through descriptive statistics. Cronbach’s alpha coefficient and item-total correlation were used to assess internal consistency and reliability. Additionally, the test-retest method was used as a measure of the stability of the scale over time. Values of Cronbach’s alpha coefficient ≥ 0.90 were considered excellent, ≥ 0.80 good, ≥ 0.70 acceptable, and ≥ 0.60 questionable. The above-mentioned values were deemed acceptable for Cronbach’s alpha coefficient [43]. For the calculation of item-total correlations and test-retest correlations, the assumptions of normality were checked, and non-parametric statistics techniques (rho of Spearman) were used for data not normally distributed [44]. A range of correlation coefficient between 0.29 and 0.90 was deemed acceptable and an r-value of 0.10 was considered low, of 0.30 medium and of 0.50 high [45, 46]. SPSS version 28 was used for performing statistical analyses.

Confirmatory Factor Analysis (CFA) was conducted to test construct validity of SSE-ITA using Mplus v.6. Prior to proceeding with this type of test, assumptions of normality were tested [47]. Initially, missing data were checked by using the Little’s Missing Completely at Random (MCAR) Test. Subsequently, the multivariate normality assumption was assessed by using the Mardia test and the most appropriate statistical technique were selected to test the model with three first-order factors (“Debriefing and Reflections”, “Clinical Reasoning,” “Clinical Learning”). Absolute and relative fit indexes comparing reproduced co-variance matrix with empirical data were adopted. The following indexes were assessed: Root Mean Square Error of Approximation (RMSEA), Standardized Root Mean Square Residual (SRMR), Comparative Fit Index (CFI), and Tucker-Lewis Index (TLI). Model fit was considered robust if RMSEA and SRMR < 0.08, CFI and TLI > 0.95 [48].

## Results

Out of 331 students, 266 have completed the SSE-ITA scale; resulting in a response rate of 80%. Specifically, 123 students were from the University of Modena and Reggio Emilia, while 143 were from the University of Parma. The gender distribution was 85.90% female and 14.10% male and the mean age was 22.69 years ± 4 years.

### Reliability analysis

Table 1 shows the main results of the reliability analysis on the SSE-ITA scale:

**Table 1:** SSE-ITA scale: reliability analysis main results (*n* = 266)

Subscale /items	Cronbach α if item deleted		Item-total correlation coefficient (rho of Spearman)	
	Subscale	Overall scale α = 0.94	Subscale	Overall scale
DR	α = 0.91			
i_1	0.90	0.94	0.76*	0.70*
i_2	0.90	0.94	0.76*	0.67*
i_3	0.91	0.94	0.74	0.71*
i_4	0.90	0.94	0.75*	0.69*
i_5	0.90	0.94	0.79*	0.74*
i_6	0.90	0.94	0.75*	0.72*
i_7	0.90	0.94	0.78*	0.71*
i_8	0.90	0.94	0.80*	0.77*
i_9	0.90	0.94	0.74*	0.71*
CR	α = 0.84			
i_10	0.78	0.94	0.80*	0.72*
i_11	0.78	0.94	0.83*	0.72*
i_12	0.78	0.94	0.80*	0.71*
i_13	0.82	0.94	0.74*	0.66*
i_14	0.83	0.94	0.67*	0.68*
CL	α = 0.86			
i_15	0.83	0.94	0.81*	0.76*
i_16	0.82	0.94	0.86*	0.73*
i_17	0.81	0.94	0.85*	0.73*
i_18	0.82	0.94	0.84*	0.72*

Note. i_: item; DR: subscale ‘’Debriefing and Reflections’’; CR: subscale “Clinical Reasoning”;

CL: subscale “Clinical Learning” *; *p* < 0.001.

As measure of reliability, Cronbach’s alpha coefficient was used showing 0.94 for the overall scale, indicating excellent reliability; the sub-scale with the highest Cronbach’s alpha was “Debriefing and Reflections” (α = 0.91), followed by “Clinical Learning” (α = 0.86) and “Clinical Reasoning” (α = 0.84). When removing a given item from the scale, no increase in the Cronbach’s alpha coefficient was noted; hence, analysis of items based on correlation led the researchers to conclude that no items needed to be excluded from the scale (Table 1).

In addition, the variables were not normally distributed: Kolmogorov-Smirnov test and Shapiro-Wilk test were significant for all items, overall score and score of each subscale (*p* < 0.001); therefore, rho of Spearman was used to test item-total correlations and test-retest correlation. A large correlation coefficient of 0.60 and above resulted between each item and its sub-scale and each item and overall scale score; they are all statistically significant (Table 1).

Table 2 shows the descriptive statistics and the test-retest correlation coefficient of each item.

**Table 2:** Test-retest reliability results

Items	Median (IQR)		Correlation Coefficient (rho of Spearman)
	Test (*n* = 266)	Retest (*n* = 104)	
i_1	5.00 (3)	5.00 (4)	0.58*
i_2	5.00 (3)	5.00 (2)	0.23*
i_3	4.00 (4)	5.00 (2)	0.43*
i_4	5.00 (4)	5.00 (2)	0.28*
i_5	5.00 (3)	5.00 (2)	0.50*
i_6	5.00 (3)	5.00 (2)	0.10*
i_7	5.00 (2)	5.00 (2)	0.42*
i_8	5.00 (3)	5.00 (1)	0.41*
i_9	5.00 (4)	5.00 (2)	0.62*
i_10	4.00 (3)	5.00 (2)	0.37*
i_11	4.00 (2)	5.00 (2)	0.37*
i_12	4.00 (4)	5.00 (3)	0.48*
i_13	4.00 (4)	5.00 (2)	0.52*
i_14	5.00 (3)	5.00 (2)	0.55*
i_15	5.00 (3)	5.00 (2)	0.62*
i_16	4.00 (3)	5.00 (4)	0.48*
i_17	4.00 (3)	5.00 (3)	0.52*
i_18	4.50 (4)	5.00 (2)	0.39*

Note. i_= item; IQR = Interquartile Range; *= *p* < 0.001

The test-retest correlation coefficient was low in item 6 (*r* = 0.10), high in items 13,14,15,17 (*r* > 0.50) and medium for the remaining items (*r* > 0.30). However, the analyses show a high percentage (> 62%) of concordance for all items in the test and retest and the medians, in the test and retest, are the same in 10 items. The high degree of homogeneity of the data does not allow an assessment of the stability of the scale over time under each condition (satisfied and not satisfied) and the values of the correlation coefficients obtained could be influenced by the latter.

### Confirmatory Factor Analysis

CFA (Confirmatory Factor Analysis) was conducted on SSE-ITA scale to test the three-factor structure of the original scale [3] and of the Italian version [31]. Missing data were less than 4% for each score and the MCAR test results were non-significant (Chi-square = 52.56, DF = 61, Sign. = 0.71), indicating that data were missing randomly without compromising the estimation in data analyses. The18 items of the SSE-ITA showed an asymmetry higher than I1.0I while the Mardia test yielded significant multivariate skewness (M = 25.38, SD = 1.14, *p* < 0.001) along with Kurtosis (M = 357.58, SD = 3.23, *p* < 0.001), Maximum Likelihood with Robust standard errors (i.e. MLR) was used as estimator in the following analysis to prevent any negative impact when dealing with non-normal data [49].

As shown in Table 3, the CFA conducted on SSE-ITA showed good fit indices confirming the factor structure found in the original study.

**Table 3:** SSE-ITA: confirmatory factor analysis results (*n* = 266)

Fit Measures	Goodness of fit	SSE-ITA Model fit information
RMSEA	Acceptable < 0.80 Good < 0.05 Excellent < 0.01	0.05
CFI	Good > 0.90 Excellent > 0.95	0.95
TLI	Good > 0.90 Excellent > 0.95	0.94
SRMR	Acceptable < 0.10 Good < 0.08	0.05

Note. RMSEA: Root Mean Square Error of Approximation; CFI: Comparative Fit Index;

TLI: Tucker- Lewis Index; SRMR: Standardized Root Mean Square Residual.

Table 4 shows the structure of SSE-ITA with items factor loading. Item loadings range from I0.64I to I0.79I; this means that items are good indicators of their respective sub-scale as they are higher than 0.45, the cut-off set by some saturation guidelines [50].

**Table 4:** Factors loadings resulting from CFA of SSE_ITA (*n* = 266)

Items	Factor 1 DR	Factor 2 CR	Factor 3 CL
i_1	0.73		
i_2	0.73		
i_3	0.70		
i_4	0.68		
i_5	0.76		
i_6	0.76		
i_7	0.76		
i_8	0.79		
i_9	0.72		
i_10		0.75	
i_11		0.78	
i_12		0.79	
i_13		0.64	
i_14		0.65	
i_15			0.79
i_16			0.76
i_17			0.77
i_18			0.79

Note. i_: item; DR: subscale ‘’Debriefing and Reflections’’; CR = subscale “Clinical Reasoning”;

CL = subscale “Clinical Learning”.

## Discussion

This study aimed at investigating the psychometric properties of the SSE-ITA scale on a larger multi-centre sample of nursing students. The study specifically tested the tool for psychometric properties such as internal consistency and structural validity.

Internal consistency is the degree of interrelatedness among the items and it is often based on Cronbach’s alpha coefficient [51]. The study results revealed that the SSE-ITA overall scale, as well as its three sub-scales, exhibits high internal consistency. In fact, in this study, the values are higher than in the first Italian validation study [31]. These results are aligned with those emerging from of other validation studies of the SSE. Particularly, in this study Cronbach’s alpha is slightly lower than in the original version of the scale in relation to subscale ‘’Debriefing and reflections’’ for which α = 0.93 and to sub-scale ‘’Clinical reasoning’’ for which α = 0.85 [3].

Compared to the Croatian version of the scale (SSE-CRO) [32], Cronbach’s alpha is slightly higher in this scale than in the SSE-ITA for the first factor CRO - F1 (α = 0.90) and the third factor CRO - F3 (α = 0.73), as well as in the overall scale (α = 0.92). In the second factor CRO-F2, the alpha coefficient (α = 0.84) remains consistent with the subscale “Clinical Reasoning” of SSE-ITA.

Recently, the Turkish version of the scale (SSES-TR) was validated as well [33]. The SSES-TR exhibits lower Cronbach’s alphas compared to SSE-ITA in both the overall scale (α = 0.93) and the sub-scales (α = 0.90 for the “Debriefing and Reflections” sub-scale, α = 0.77 for the “Clinical Reasoning” sub-scale, and α = 0.81 for the “Clinical Learning” sub-scale). The CFA conducted on the SSES-TR also indicates acceptable or good fit measures (RMSEA = 0.09, CFI = 0.98, TLI = 0.98, SRMR = 0.09).

Therefore, the results of the CFA suggest that the scale possesses good structural validity; this means that the degree to which the instrument scores reflect the dimensionality of satisfaction as the construct is adequate [51]. The main satisfaction indicators in the original scale were drawn from the literature and a panel of experts subsequently reached consensus on their related items; a large correlation between each item and its subscale and items and the overall scale was found. Therefore, these variables may contribute to better defining what satisfaction in simulation really means and promoting research in this area.

Satisfaction holds a primary position in Kirkpatrick’s educational program evaluation model, specifically focusing on perceptions. This aspect becomes crucial when examining the impact of simulation-based teaching programs, particularly in the context of the uneven integration of high-fidelity simulation in Italian educational programs. There are prevailing misconceptions and low expectations among students in this regard [52].

To enhance the generalizability of validation results for the Italian version of the instrument, our study involved multiple centres and students from both first and third academic years. This is a notable improvement compared to the initial validation study, which included only second-year students from a single centre. Future studies using this tool can provide valuable insights into its validation.

Using a validated tool for measuring satisfaction is essential not only for the Italian context but also for addressing gaps in the literature. Two recent meta-analyses have reported non-significant results for student satisfaction in high-fidelity simulation, possibly influenced by factors such as simulation-related elements and the number of exposures [13, 20, 53]. Interestingly, repeated exposures have been shown to enhance student satisfaction [27, 52].

A critical consideration is the comparison among different types of simulation and the identification of factors influencing both learning outcomes and satisfaction. Studies assessing these aspects using validated tools are particularly desirable, especially with the advancements in robotics and artificial intelligence, which are reshaping educational standards [52].

In light of our study findings, the Satisfaction with SSE scale proves to be a valuable assessment tool in Italian simulation settings. When translated, it holds potential for international use, enabling comparability. It’s worth noting that satisfaction significantly impacts student retention [54] and confidence, influencing nurses’ behaviors in professional settings [55].

For future research, it’s crucial to acknowledge the limitations of this study. The homogeneity of data between test and retest poses a limitation, influencing the statistical analyses for the time stability measure of the scale. Additionally, the wide time interval (from 4 to 42 days) for retests may have influenced the obtained results [56].The previous validation study of the SSE-ITA indicated good test-retest reliability of the scale [31]. Similarly, the test-retest reliability coefficients of SEES-TR were found to be comparable [33].

Another notable limitation is the absence of measurements for convergent and cross-cultural validity. Convergent validity assesses whether the scores of the tested tool align with expectations when correlated with other tools [51]. For instance, Tüzer et al. [33] compared the SSES-TR with “The Scale of Student Satisfaction and Confidence In Learning” to establish convergent validity. Cross-cultural validity, on the other hand, gauges how well the performance of items on a translated or culturally adapted instrument reflects that of the original version [51]. To address this psychometric property, a combined dataset of scores from Italy and another country with comparable samples could provide valuable insights.

## Conclusions

In conclusion, satisfaction plays a pivotal role in achieving learning outcomes in simulation. The SSE-ITA scale, with demonstrated validity and reliability, stands as a valuable tool for assessing simulation in Italian nursing students. Widespread use of this scale and its validated versions can facilitate satisfaction assessments in diverse contexts and support evidence of its psychometric integrity, particularly in cross-cultural validity. This opens avenues for further studies investigating the relationship between satisfaction and learning outcomes in simulation.

## Data Availability

The datasets used and/or analysed during the current study are available from the corresponding author on reasonable request.

## Abbreviations

SSE-ITA: Satisfaction with Simulation Experience - Italian Version
I-CVI: Item-Content Validity Index value
S-CVI: Subscale-Content Validity Index
CFA: Confirmatory Factor Analysis
RMSEA: Root Mean Square Error of Approximation
SRMR: Standardized Root Mean Square Residual
CFI: Comparative Fit Index
TLI: Tucker-Lewis Index
DR: subscale 1 “Debriefing and Reflections”
CR: subscale 2 “Clinical Reasoning”
CL: subscale 3 “Clinical Learning”
